# A STAT3-NFkB/DDIT3/CEBPβ axis modulates ALDH1A3 expression in chemoresistant cell subpopulations

**DOI:** 10.18632/oncotarget.3703

**Published:** 2015-03-30

**Authors:** Claudia Canino, YuYing Luo, Paola Marcato, Giovanni Blandino, Harvey I. Pass, Mario Cioce

**Affiliations:** ^1^ Division of Thoracic Surgery, Department of Cardiothoracic Surgery, Langone Medical Center, New York University, New York, USA; ^2^ New York University School of Medicine, New York, USA; ^3^ Department of Pathology and Department of Microbiology & Immunology, Dalhousie University, Halifax, Nova Scotia, Canada; ^4^ Translational Oncogenomics Unit, Italian National Cancer Institute ‘Regina Elena’, Rome, Italy; ^5^ Department of Oncology, Juravinski Cancer Center-McMaster University, Hamilton, Ontario, Canada

**Keywords:** ALDH, STAT3, NFkB, DDIT3, CEBPβ

## Abstract

Here we studied the relevance and modulation of aldehyde dehydrogenase (ALDH) expression in malignant pleural mesothelioma (MPM) chemoresistant cell subpopulations (ALDH^bright^ cells), which survive pemetrexed + cisplatin treatment in vitro and in vivo. Expression of the ALDH1A3 isoform was invariably enriched in purified ALDH^bright^ cells from multiple MPM cell lines and accounted for the enzymatic activity of those cells. RNAi mediated downregulation of ALDH1A3 reduced the survival of the ALDH^bright^ cells at steady state and, much more, after pemetrexed + cisplatin treatment. We demonstrated, for the first time, that a pSTAT3(tyr705)-NFkB(p65) complex is required for the repression of DDIT3 mRNA and this ensures high levels of CEBPβ-dependent ALDH1A3 promoter activity. Inhibition of STAT3-NFkB activity allowed high levels of DDIT3 expression with increased formation of a DDIT3-CEBPβ complex. This reduced the occupancy of the ALDH1A3 promoter by CEBPβ, thus largely reducing the ALDH1A3 expression. Consequently, survival of ALDH^bright^ cells in pemetrexed + cisplatin-treated cultures was impaired, following increased apoptosis. We show that such a mechanism is relevant *in vivo* and underlies the action of butein, a dual STAT3-NFkB inhibitor capable of abating the chemoresistance of mesothelioma cells *in vivo*. The possible broad translational relevance of the described mechanism is discussed.

## INTRODUCTION

Malignant Pleural Mesothelioma (MPM) is a neoplastic disease whose challenging clinical management is characterized by silent progression, extremely low response rate to chemotherapy (pemetrexed + cisplatin) and poor prognosis [1-3]. There is an unmet need for better therapeutic options for such a fatal disease. We have recently shown that pemetrexed and cisplatin treatment of MPM cell lines and primary cultures triggered the emergence of cell subpopulations exhibiting absolute chemoresistance, mesenchymal traits and high levels of aldehyde dehydrogenase (ALDH) activity (ALDH^bright^ cells) [4] and those properties were shared by lung cancer ALDH^bright^ cells resistant to gefinitib and cisplatin [5]. The ALDH^bright^ cells represented, quantitatively, the main chemoresistant cell subpopulation in several other tumors and could be tracked by FACS-based assays [4, 6-8]. ALDHs are a family of enzymes with heterogeneous intracellular localization and substrate specificity, which function by oxidizing intracellular aldehydes to carboxylic acid, in physiological and patho-physiological conditions [9, 10]. Additionally, ALDHs have been involved in conferring resistance to some alkylating agents [11, 12]. Enriched expression of ALDH isoforms has been observed in a conspicuous number of developmentally unrelated tumors [13-17]. For example, expression of the ALDH1A3 has been experimentally shown to modulate survival of melanoma and glioma cell subpopulations exhibiting properties of cancer stem cells [18, 19] and to promote pro-tumorigenic features in breast cancer cells [20]. Therefore, ALDHs can be therapeutically relevant targets in cancer [6].

Members of the CAAT/Enhancer-Binding Protein (CEBP) family were shown to modulate expression of the ALDH enzymes in different experimental settings, through binding to conserved CAAT binding site in proximity of the transcription start site [21-24]. On the other hand, the DDIT3/CHOP/GADD153 gene, whose levels are modulated by a plethora of stress stimuli [25], including chemotherapy [26, 27], was shown to modulate the CEBPβ transcriptional activity via protein-protein interactions in fibroblasts [25] and retinoic acid (RA) treated cells [22]. Notably, DDIT3 was shown to be modulated by STAT3 [28, 29].

The STAT3 pathway may modulate the number of NSCLC- and mesothelioma- ALDH^bright^ cells [4, 30] and, notably, glioma cells of the mesenchymal subtype, which require STAT3 (and CEBPβ) for their survival [31], exhibited high levels of ALDH1A3 expression [19]. NFκB is constitutively active in most cancers [32] and exhibits extensive networking with several cancer signaling pathways, including STAT3 [33, 34]. We have shown that STAT3 and NFkB physically and functionally interacted in chemotherapy resistant MPM cell lines [35]. Treatment of MPM cell lines with butein (a multifunctional tetrahydroxychalcone), interfered with the stability of the STAT3-NFkB and this correlated with decreased chemoresistance *in vitro* and *in vivo* [35]. However, the molecular mechanism whereby interference with the STAT3-NFkB complex could affect MPM chemoresistance were not defined. Additionally, it was unclear whether this involved rearrangement of chemoresistant MPM cell subpopulations. Last but not least, it was not known which ALDH isoform(s) were expressed in the chemoresistant ALDH^bright^ MPM cells and the functional relevance of ALDH expression for those cell subpopulations.

By combining *in vitro* and *in vivo* approaches, we demonstrate here that a STAT3-NFkB-dependent repression of DDIT3 expression ensures high levels of CEBPβ-dependent ALDH1A3 expression and that modulates the survival and resistance of the ALDH^bright^ cells to pemetrexed + cisplatin treatment *in vitro* and *in vivo*. Thus, we show that repression of DDIT3 levels may represent a cell subpopulation-specific mechanism of resistance to therapy which can be targeted *in vivo* by butein. Given the broad alteration of the STAT3 and NFkB signaling pathways in cancer [32, 36] and the presence of ALDH^bright^ cells in many neoplastic diseases, the conclusion of this study may be of broader relevance.

## RESULTS

### Butein affects the survival of ALDH^bright^ cells after pemetrexed + cisplatin treatment

**Figure 1:**
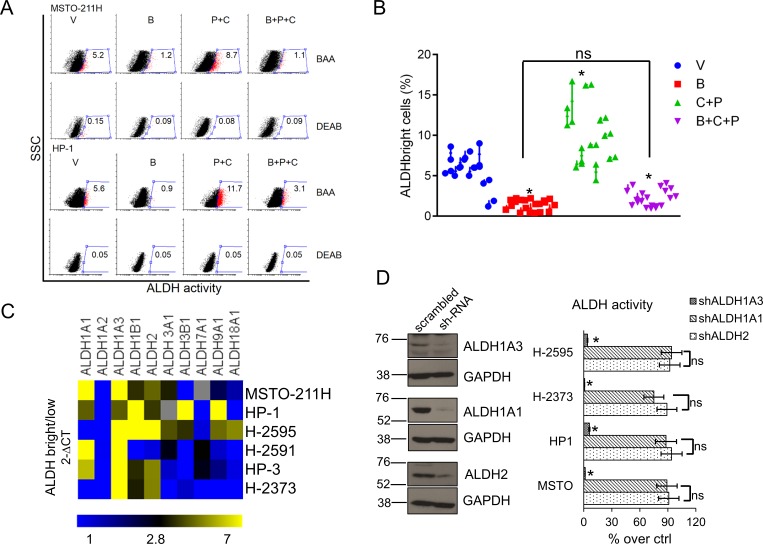

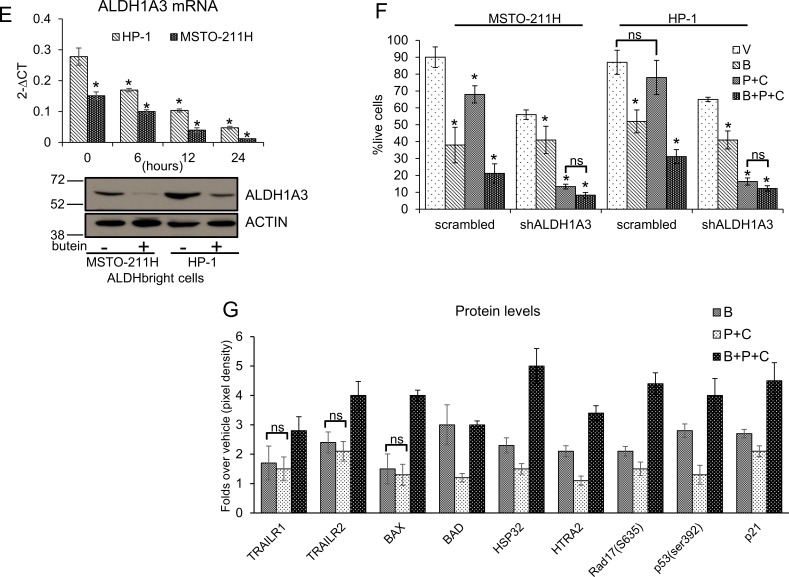
Butein affects the survival of MPM chemoresistant cell subpopulations (ALDH^bright^ cells) **A**. Butein reduces the number of ALDH^bright^ cells in MPM cultures. Representative flow cytometry plots showing the percentage of ALDH^bright^ cells (red, gated) in MPM cell cultures treated for 24hrs with vehicle (V: DMSO 0.05%) and butein (B: 18μM), alone or in combination with pemetrexed + cisplatin (P+C: 10 μM + 5 μM, respectively) and stained for ALDH activity at 96hrs. The percentage of ALDH^bright^ cells was determined over the same cells treated with a specific ALDH inhibitor (DEAB) immediately after adding the ALDH substrate (BAA). **B**. Graph showing the average ALDH^bright^ cell number from the grouped MPM cell lines (n=10) treated as indicated in 1A. C-D. The ALDH1A3 is responsible for the aldehyde dehydrogenase (ALDH) activity of the ALDHbright cells. **C**. Heat map: mRNA levels of the detectable ALDH isoforms in purified ALDH^bright^ and ALDH^low^ cells from 6 MPM cell lines. **D**. Left. Representative western blotting of MSTO-211H cells infected with a pool of ALDH1A1, ALDH2 and ALDH1A3 targeting shRNAs and control (scrambled) shRNAs, selected with puromycin and stained as indicated. Right. ALDH activity in 4 representative MPM cell lines infected as indicated in the left panel. Percentage of ALDH activity is relative to cells infected with the scrambled shRNA (control). Duplicate experiments. **E**. Butein modulates the expression of ALDH1A3. Upper. ALDH1A3 mRNA levels of purified MSTO-211H and HP-1 ALDH^bright^ cells treated with butein for the indicated times, as assessed by quantitative PCR. Lower. Western Blotting with anti-ALDH1A3 specific antibodies and anti-actin (as a loading control) of whole cell lysates from purified MSTO-211H and HP-1 ALDH^bright^ cells treated with butein for 36hrs **F**-**G**. Butein treatment affects the viability of purified ALDHbright cells. Percentage of SYTOX red negative cells from MSTO-211H and HP-1 ALDH^bright^ cells infected with a vector expressing scrambled shRNA or ALDH1A3-shRNAs, respectively) and treated as indicated for 24hrs and harvested at 72hrs. **G**. Protein levels of stress response genes and apoptotic effectors in the indicated MPM cell lines treated as in fig. 1F and harvested at 48hrs. Duplicate experiments. Histogram bars represent the mean ± s.e.m of ≥ three experiments, except were indicated otherwise. Statistics: * *p* < 0.05; ns=not significant: (*p* > 0.05). One-way analysis of variance with Tukey's *post hoc* corrections-comparing the mean of each group with the mean of every other group (B) or Student's t-test (comparing each sample to its control or, when indicated, to other samples within the same group) (D, E. F. G).

Given that the ALDH^bright^ MPM cells are the main subcellular population resistant to pemetrexed [4] and given the ability of butein to counteract the chemoresistance of MPM cells *in vitro* and *in vivo* [35], we tested the hypothesis that the latter compound may affect the survival of the ALDH^bright^ cell subpopulations. Treatment with butein (B: 18μM), alone or in combination with pemetrexed + cisplatin (P+C: 10 μM + 5 μM, respectively) for 96hrs strongly reduced the number of ALDH^bright^ cells in multiple unrelated MPM cell lines (n=10) and prevented their increase after pemetrexed+cisplatin (P+C) treatment (Fig. 1A-1B, *p* < 0.05). Since disappearance of ALDH^bright^ cells may follow direct enzyme inhibition or downregulation of ALDH expression, we explored which of the two processes underlied the effects of butein. Short term (0-12hrs) treatment of MPM cells with butein did not affect the ALDH activity (suppl. Fig. 1A, upper and lower). To assess whether butein may modulate the expression rather than the activity of ALDHs, we first determined which ALDH isoform(s) would be enriched in the ALDH^bright^ cells (Fig. 1C). We assessed (by quantitative PCR) the mRNA levels of the (detectable) ALDH isoforms in FACS sorted ALDH^bright^ and ALDH^low^ cells from unrelated MPM cell lines (average purity of the ALDH^bright^ cells: 92-96%, n=6). Quantitative PCR revealed that the ALDH1A3 (and, to a much lesser extent, ALDH1A1 and ALDH2) was enriched in the ALDH^bright^ cells of all the analyzed cell lines (*p* < 0.05) (Fig. 1C, heat map).

### The ALDH1A3 isoform is responsible for the ALDH activity of the MPM cells

Next, we infected MSTO-211H and HP-1 cells with shRNAs against ALDH2, ALDH1A1 and ALDH1A3, respectively (Fig. 1D, left). We found that only knocking down of ALDH1A3 correlated with a reduction of the ALDH^bright^ cell number (Fig. 1D, right). Together this data (Figs. 1C and 1D) suggests that the ALDH1A3 isoform is primarily responsible for ALDH activity of MPM cells.

### Butein downregulates the expression of ALDH1A3 thereby affecting the viability of the MPM ALDH^bright^ cells

Quantitative PCR analysis of RNA extracted from FACS sorted ALDH^bright^ cells revealed that butein treatment triggered a strong, time dependent, downregulation of the of ALDH1A3 mRNA levels (Fig. 1E, upper panel), in agreement with our hypothesis that butein affects the expression rather than the activity of the ALDH enzyme(s). This paralleled a sharp decrease, in the same cells, of the protein levels (lower panel, Fig. 1E). To detail the fate of ALDH^bright^ cells treated with butein and its relationship with the levels of ALDH1A3, we evaluated the viability of MSTO-211H and HP-1 cells treated with vehicle (V), butein (B), in absence or presence of pemetrexed + cisplatin treatment (P+C vs B+P+C, respectively) and upon RNAi-mediated downregulation of ALDH1A3 (Fig. 1F). SYTOX red staining revealed that the (P+C) treatment marginally affected the viability of the control vector-infected cells (as compared to the vehicle-treated cells), in line with the increased resistance of the ALDH^bright^ cells to these treatments [4]. Co-treatment with butein (B+P+C) strongly increased the effect of the P+C treatment on the same cells (Fig. 1F). In the same conditions, downregulation of ALDH1A3 strongly mimicked the effect of butein and did not significantly increase the effect of butein treatment (Fig. 1F), suggesting that modulation of ALDH1A3 levels is the main mechanism mediating the effect of butein on the viability of the ALDH^bright^ cells. Probing of an apoptosis antibody array with whole cell lysates from HP-1 ALDH^bright^ cells treated with B, P+C or with B+P+C confirmed increased levels, in the B+P+C treated cells (as compared to B and P+C treated samples-*p* < 0.05), of multiple apoptotic effectors: TRAILR1 (DR4) and TRAILR2 (DR5); BAX and BAD and the mitochondria-released HTRA2 (Fig.1G). Notably, some DNA damage and stress response genes were also upregulated with a similar trend (Fig. 1G).

### Butein downregulates the activity of the ALDH1A3 promoter

**Figure 2:**
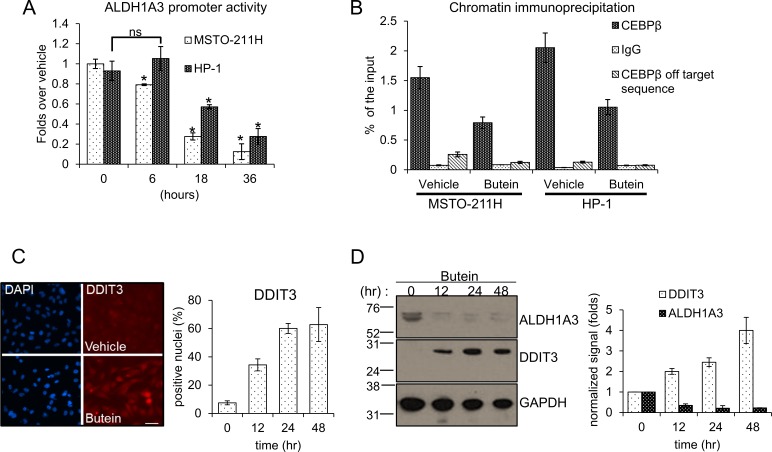

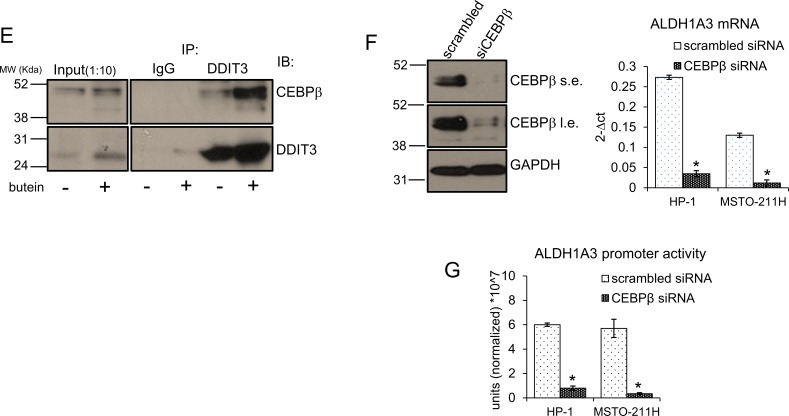
**A**. Butein modulates the ALDH1A3 promoter activity by modulating DDIT3 levels. Normalized luciferase activity of MSTO-211H and HP-1 cells transfected with a ALDH1A3-luciferase expressing vector and treated with butein (18 μM) at the indicated times. **B**. Butein treatment reduces the occupancy of the ALDH1A3 promoter by CEBPβ. Quantitative PCR. Amplification of the CEBPβ binding region from chromatin immunoprecipitated with anti-CEBPβ and control rabbit IgG from MSTO-211H and HP-1 cells treated with vehicle or butein (18 μM), respectively, for 18 hours. Percentage of enrichment relative to the input chromatin is reported. Amplification of a DNA sequence not containing the CEBPβ binding site was used as an “off target” control to probe the anti-CEBPβ immunoprecipitated material. C-D. Butein affects DDIT3 protein levels. **C**. Left. Representative fluorescence micrographs of MSTO-211H cells treated with vehicle or butein (18 μM), for 24hrs and stained with anti-DDIT3 antibodies (right). Cell nuclei were stained with DAPI (left). A minimum of 8 fields (containing ≥40 nuclei) was counted in duplicate experiments. Scale bar: 20μm. Right. Histograms showing the average percentage of DDIT3 positive nuclei from duplicate experiments. **D**. Left. Western blotting of whole cell lysates from MSTO-211H cells treated with butein (18 μM), as indicated and stained with anti-DDIT3, anti-ALDH1A3 and anti-GAPDH antibodies (as a loading control). Right. Histograms showing the changes in intensity signal of DDIT3 and ALDH1A3 (normalized to actin with Image J software). **E**. Increased interaction of DDIT3 and CEBPβ in butein-treated cells. Western Blotting with anti-DDIT3 and anti CEBPβ antibodies of whole cell lysates immunoprecipitated with anti-DDIT3 antibody and isotype matched mouse IgG (as a control), respectively. **F**. RNAi-mediated downregulation of CEBPβ mimicks the effects of butein on ALDH1A3 expression. Left. Western blotting with anti- CEBPβ antibodies of MSTO-211H and HP-1 cells transfected with control (scrambled) and CEBPβ-targeting siRNA. (s.e: short exposure; l.e.: long exposure). GAPDH used as a loading control. Right. mRNA levels of ALDH1A3 in HP-1 and MSTO-211H cells transfected with scrambled or CEBPβ-targeting siRNAs assessed by quantitative PCR. **G**. Luciferase activity of HP-1 and MSTO-211H cells transfected with an ALDH1A3 luciferase expression vector and, 24hrs later, with scrambled or CEBPβ-targeting siRNAs. Histogram bars represent the mean ± s.e.m of ≥ three experiments, except were otherwise indicated. Statistics: * *p* < 0.05; ns=not significant: (*p* > 0.05). Student's t-test (comparing each sample to its control or, when indicated, to other samples within the same group).

To investigate the modulation of ALDH1A3 mRNA by butein (Fig. 1E), we transfected MSTO-211H and HP-1 MPM cells with a luciferase reporter vector containing the ALDH1A3 promoter (from −900 to +170bp). Treatment of the cells with vehicle or butein 24hrs later revealed that the latter strongly downregulated the luciferase expression in a time dependent way (Fig. 2A). This experiment suggested that butein may directly affect the ALDH1A3 mRNA levels by modulating its promoter activity.

### Butein treatment reduces the binding of CEBPβ to the endogenous ALDH1A3 promoter

A CAAT box (a consensus for the binding of the CEBPβ transcription factor), was shown to be crucial for the promoter activity of several ALDH family members [21, 22] and in house bioinformatics analysis (MATCH^TM^, BIOBASE) revealed that the ALDH1A3 promoter contained a conserved CAAT box (−50/−36bp from the transcription start site). Quantitative PCR analysis of chromatin immunoprecipitation (CHIP) with anti-CEBPβ antibodies from MSTO-211H and HP-1 cells revealed effective occupancy of the CEBPβ- binding site in vehicle-treated cells (as compared to a isotype-matched rabbit IgG). Butein treatment strongly reduced the occupancy of the CEBPβ binding site by CEBPβ (Fig. 2B). This strongly correlated with the effect of the drug on the ALDH1A3mRNA and protein levels (Fig. 1E). CEBPβ transcriptional activity is modulated through protein-protein interactions involving several transcription factors, including the stress response DDIT3/CHOP/GADD153, formerly identified as both a modulator of CEBPβ activity and a stress responsive factor (including chemotherapy) [25].

### Butein affects DDIT3 mRNA and protein levels by modulating the DDIT3 promoter

Thus, we investigated whether butein treatment may alter the levels of DDIT3 and whether this may interfere with CEBPβ activity. Butein treatment increased the levels of DDIT3 mRNA over time in two representative MPM cell lines (Suppl. Fig. 2) and this matched increased levels of the protein, as shown by indirect immunofluorescence and by western blotting (Fig. 2C-D). Additionally, increased levels of DDIT3 matched decreased amount of ALDH1A3 in the treated cells (Fig. 2D).

### Increased binding of DDIT3 to CEBPβ in butein-treated cells

Given the ability of DDIT3 to negatively modulate CEBPβ transcriptional activity via protein-protein interactions [22, 25], we immunoprecipitated DDIT3 from unfractionated extracts of vehicle- and butein-treated MSTO-211H (and HP-1) cells (Fig. 2E). Western blotting of the immunoprecipitated material readily detected increased amounts of CEBPβ bound to DDIT3 upon butein treatment, suggesting that the increased DDIT3 in the butein treated cells interacted more or more strongly with CEBPβ (Fig. 2E). This strictly correlated with the observed decreased amount of CEBPβ bound to the ALDH1A3 promoter (Fig 2B) and, ultimately, with the reduced levels of ALDH1A3 mRNA, in the butein treated samples (Fig. 1E). RNAi-mediated downregulation of CEBPβ (Fig. 2F, left) strongly decreased both the ALDH1A3 endogenous mRNA (Fig. 2F, right) and the luciferase activity driven by the ALDH1A3 promoter (Fig. 2G), thereby strictly mimicking the effect of butein and providing further support to the previous observations.

### STAT3 inhibition underlies the effect of butein on the DDIT3 levels

**Figure 3:**
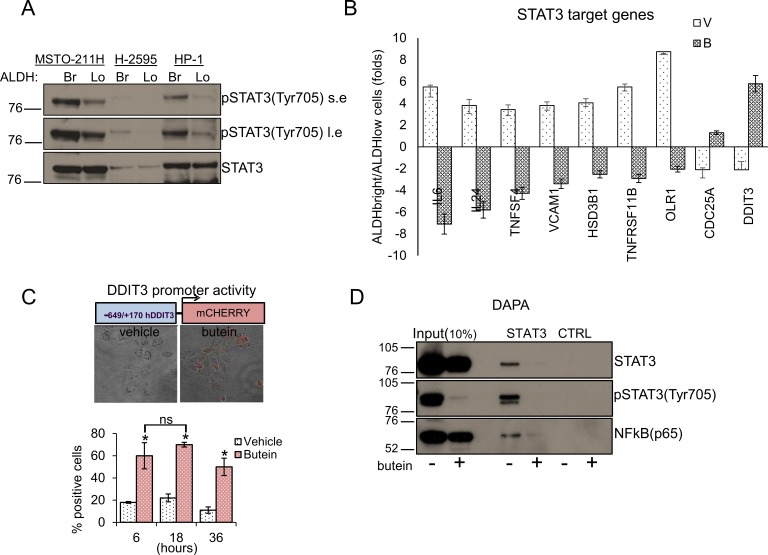

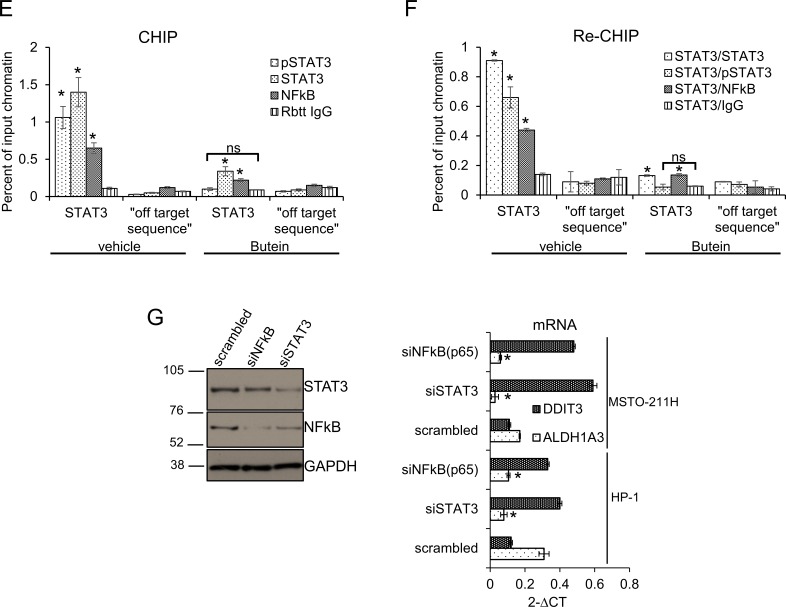
STAT3 inhibition underlies the effect of Butein on the DDIT3 levels **A**. ALDHbright cells exhibit increased activation of the STAT3 pathway. Western blotting with specific anti-STAT3 and anti-phospho-STAT3(tyr705) antibodies of whole cell lysates from purified ALDH^bright^ and ALDH^low^ of three representative MPM cell lines (s.e: short exposure; l.e.: long exposure). **B**. mRNA levels of multiple STAT3 target genes in MSTO-211H ALDH^bright^ vs ALDH^low^ cells, upon treatment with vehicle or butein (18 μM) for 24hrs. **C**. Upper. Combined bright field + fluorescent micrographs of MSTO-211H cells transfected with a mCherry reporter driven by the minimal DDIT3 promoter (−649/+170) and treated with butein (18 μM) for 6hrs. Scale bar: 20 μm. Lower. Percentage of mCherry positive cells in butein-treated cell cultures. A minimum of 8 fields (containing ≥30 cells) was counted in duplicate experiments. D-F. Butein affects the binding of STAT3 and NFkB to the DDIT3 promoter. **D**. DNA Affinity Precipitation assay (DAPA) with a biotinylated oligonucleotide containing either a STAT3 binding site in the DDIT3 promoter (STAT3) or a control sequence (CTRL), respectively. Western blotting of the DAPA-eluted from nuclear extracts of MSTO-211H cells treated with vehicle and butein (18 μM, 6hrs). Staining with antibodies against pSTAT3(Tyr705), STAT3 and NFkB(p65), respectively. **E**. *In vivo* occupancy of the DDIT3 promoter. Chromatin immunoprecipitation assays. Quantitative PCR revealing enrichment for the STAT3 containing DDIT3 promoter fragment in the eluate of STAT3, pSTAT3 and NFkB immunoprecipitates from vehicle or butein-treated MSTO-211H cells (18 μM, 20hrs). A rabbit IgG and a “off target” DNA region in the same promoter were used to control for the specificity of immunoprecipitation and of the PCR reaction, respectively. **F**. RE-CHIP assays. Chromatin eluted from STAT3 immunoprecipitated material of vehicle- and butein –treated MSTO-211H cells (as from 3E) was re-immunoprecipitated with a rabbit IgG, STAT3, pSTAT3 and NFKB antibodies, respectively. Quantitative PCR revealed specific amplification of the DDIT3 promoter fragment suggesting the existence of a STAT3-NFKB complex. Duplicate experiments. **G**. RNAi-mediated downregulation of STAT3 and NFkB mimicked the effects of butein on DDIT3 and ALDH1A3 mRNA levels. Left. Western blotting with anti-STAT3 and anti-NFkB antibodies of whole cell lysates from MSTO-211H and HP-1 cells transfected with control (scrambled), STAT3 and NFkB targeting siRNA revealed effective downregulation of the protein levels. Actin used as a loading control. Right. Quantitative PCR revealed higher levels of DDIT3 mRNA and reduced levels of ALDH1A3 mRNA in the cells with reduced expression of STAT3 and NFkB. Values expressed as folds over controls (scrambled siRNAs). Statistics: * *p* < 0.05; ns=not significant: (*p* > 0.05). Student's t-test (comparing each sample to its control).

DDIT3 is a target gene of STAT3 and its levels are upregulated in cells where binding of STAT3 to its promoter is diminished [28], implying active repression. Since we and others have shown that butein inhibits STAT3 [35, 37], we tested whether butein increased the levels of DDIT3 by inhibiting STAT3 activation. First, we evaluated the status of the STAT3 pathway in purified ALDH^bright^ cells. Western blotting of whole cell lysates from both ALDH^bright^ and ALDH^low^ cells of three representative cell lines revealed strong enrichment for the pSTAT3(tyr705) signal (with slight changes in the levels of the total STAT3 protein) in the ALDH^bright^ cell fraction (Fig. 3A). Accordingly, quantitative PCR of 30 representative, literature selected STAT3 target genes [38] revealed that most of the targets exhibited higher levels in the ALDH^bright^ cells as opposed to the ALDH^low^ cells (suppl. Fig. 3). Butein treatment modulated the levels of most of the targets in both ALDH^bright^ and ALDH^low^ cells (Suppl. Fig. 3), and, to a much higher extent, the levels of a subset of those genes in the ALDH^bright^ cells, including DDIT3 (as compared to the ALDH^low^ cells) (Fig. 3B). Thus, the MPM ALDH^bright^ cells exhibited higher activation of the STAT3 pathway and responsivity to butein treatment.

We next investigated in detail the modulation of DDIT3 by butein. Since upregulation of DDIT3 by butein may result from increased promoter activity, we transfected HP-1 and MSTO-211H cells with a mCHERRY reporter driven by the minimal promoter of DDIT3 (−649/+136) [39] (Fig. 3C). Butein treatment (18 μM) strongly induced the promoter activity over time (as compared to vehicle treatment), as evidenced by the increase in the mCHERRY positive cells detected by fluorescence microscopy (Fig. 3C, p < 0.05). We next performed DNA affinity precipitation assays (DAPA) by using a biotinylated oligonucleotide containing the STAT3 binding site in the DDIT3 promoter. Western Blotting of the eluted material revealed that the binding of STAT3 to the promoter fragment *in vitro* was strongly reduced by butein treatment (18 μM for 8hrs), as compared to vehicle treatment (Fig. 3D). Staining with a phospho-STAT3 antibody (Tyr705) showed a strong reduction of the STAT3 phosphorylation in the butein-treated samples (Fig. 3D). Additionally, western blotting of the DAPA eluate with anti-NFkB(p65) antibodies revealed binding of NFkBp65 to the STAT3 oligonucleotide, which was strongly reduced upon butein-treatment (Fig. 3D). Altogheter, this correlated with the modulation of DDIT3 levels in the butein treated cells (Suppl. Fig. 2 and Fig. 2C-2D).To support these *in vitro* observations, we performed CHIP experiments. First, we immunoprecipitated the chromatin from vehicle and butein treated MSTO-211H cells (18 μM for 20hrs) with antibodies specific for STAT3, phosphoSTAT3 (tyr705) and NFkB (p65) (Fig. 3E). Notably, the phospho-STAT3 antibody does not recognize un-phosphorylated STAT3. Quantitative PCR of the eluted chromatin with primers amplifying a region encompassing the STAT3 binding sequence used for the *in vitro* binding studies, revealed specific enrichment of the DDIT3 promoter fragment in the STAT3, pSTAT3 and NFkB immunoprecipitated chromatin (as compared to control IgG immunoprecipitation) and no amplification of a “off target” region was observed (Fig. 3E). Butein treatment significantly decreased the occupancy of the STAT3 binding site by all three factors (*P* < 0.05) (Fig. 3E). This suggest that a complex containing STAT3, pSTAT3 and NFkB binds to the STAT3 binding site of the DDIT3 promoter region *in vivo*.

In order to verify the existence of such a complex, we performed sequential chromatin immunoprecipitation experiments (re-CHIP) (Fig. 3F). After a first round of CHIP with anti STAT3 antibodies, we re-immunoprecipitated the eluted material with rabbit IgGs, STAT3, pSTAT3 and NFkB antibodies, respectively. Quantitative PCR revealed enrichment of the DDIT3 promoter fragment in the secondary immunoprecipitations (Fig.3F) demonstrating a physical association of STAT3 and NFkB. Additionally, the data suggested that the complex contained STAT3 in both its un-phosphorylated and phosphorylated (Tyr705) form (Fig. 3F). In order to assess the relevance of the single factors within the complex, we knocked-down STAT3 and NFkB (p65) in MSTO-211H and HP-1 cells by using RNAi. Western blotting with anti-STAT3 and anti-NFkB antibodies revealed effective downregulation of STAT3 and NFkB protein in the transfected cells (as compared to their control-scrambled RNA (Fig. 3G left panel). Quantitative PCR revealed higher levels of DDIT3 mRNA and reduced levels of ALDH1A3 mRNA in the cells with reduced expression of both STAT3 and NFkB (Fig.3G, right panel), matching the observed binding of both factors the DDIT3 promoter *in vivo* (Fig. 3E-3F). Altogether, these data supported the reported ability of butein to interfere with the STAT3-NFkB interaction [35] and the reported indirect modulation of the DDIT3 levels by NFkB [40].

### Butein unlocks the constitutive, STAT3-dependent repression of DDIT3 mRNA in the ALDH^bright^ cells thereby affecting their tolerance to chemotherapy-induced stress

**Figure 4:**
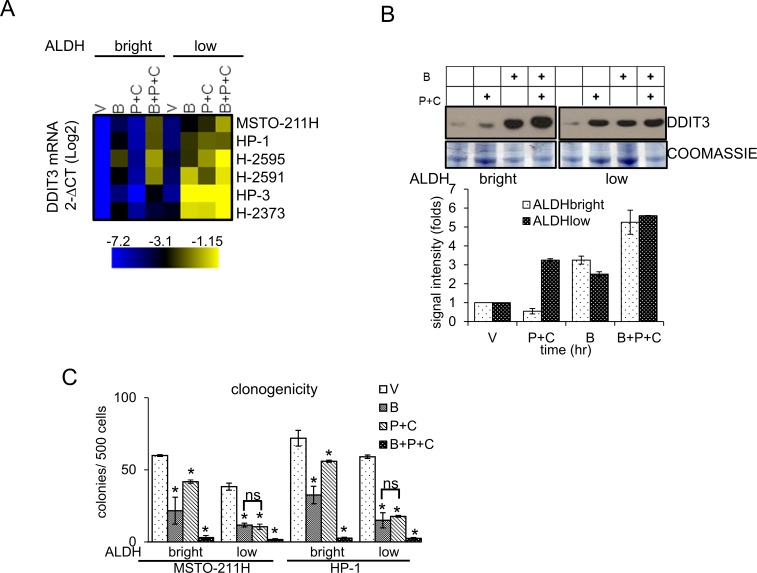
Butein unlocks the repression of DDIT3mRNA in the chemoresistant ALDH^bright^ cells **A**. Heat map. DDIT3 mRNA levels in ALDH^bright^ and ALDH^low^ cells purified from the 6M PM cell lines and treated with vehicle (V: DMSO 0.05%) and butein (B: 18 μM), alone or in combination with pemetrexed + cisplatin (P+C: 10 μM + 5 μM, respectively) for 16hrs. **B**. Upper. Western blotting with DDIT3 antibodies of whole cell lysates from purified ALDH^bright^ and ALDH^low^ MSTO-211H cells treated as in 4A. Coomassie staining used as a loading control. Lower. Histograms showing the changes in intensity signal of DDIT3 from duplicate experiments (normalized to two reference protein bands in the coomassie stained gel with Image J software). **C**. Clonogenic assays. Number of formed colonies from purified cell subpopulations of MSTO-211H and HP-1 cells treated with butein for 16hrs before seeding at clonal density. Histogram bars represent the mean ± s.e.m of triplicate experiments. Statistics: * *p* < 0.05; ns=not significant: (*p* > 0.05). Student's t-test (comparing each sample to its control or, when indicated, to other samples within the same group).

Next, we focused on modulation of the DDIT3 mRNA levels in chemotherapy treated cells, with and without butein treatment. We found that purified ALDH^bright^ cells from all the MPM cell lines tested (n=6) exhibited lower levels of DDIT3 mRNA than their ALDH^low^ counterparts (Fig. 4A, heat map). Pemetrexed + cisplatin (P+C) treatment failed to upregulate the DDIT3 mRNA in the ALDH^bright^ cells (while readily doing so in the ALDH^low^ cells), in line with the relative resistance of the ALDH^bright^ cells to P+C treatment (Fig. 4A, heat map). Importantly, butein treatment increased DDIT3 mRNA in both ALDH^bright^ and ALDH^low^ cells, raising the DDIT3 mRNA levels even in the P+C treated ALDH^bright^ cells (Fig. 4A, heat map). The described trend in the mRNA levels was similarly observed when the DDIT3 protein levels were assessed in western blottings from representative ALDH^bright^ and ALDH^low^ MSTO-211H cells (Fig. 4B). In line with the previous observations, analysis of the clonogenicity of the ALDH^bright^ and ALDH^low^ cells treated as from Fig. 4A revealed that butein treatment potentiated the P+C treatment in both cell subpopulations (Fig. 4C), suggesting that butein-mediated unlocking of the DDIT3 levels in the ALDH^bright^ cells was biologically relevant. Altogether, this correlated with the increased apoptotic response of the ALDH^bright^ cells when treated with B+P+C (Fig. 1G). Thus, butein treatment could reverse, *in vitro*, the resistance of ALDH^bright^ cells by counteracting the hyperactivation of the STAT3 pathway in the latter cell subpopulation.

### Butein treatment affects the ALDH^bright^ cell number *in vivo* and inhibits tumor growth

**Figure 5:**
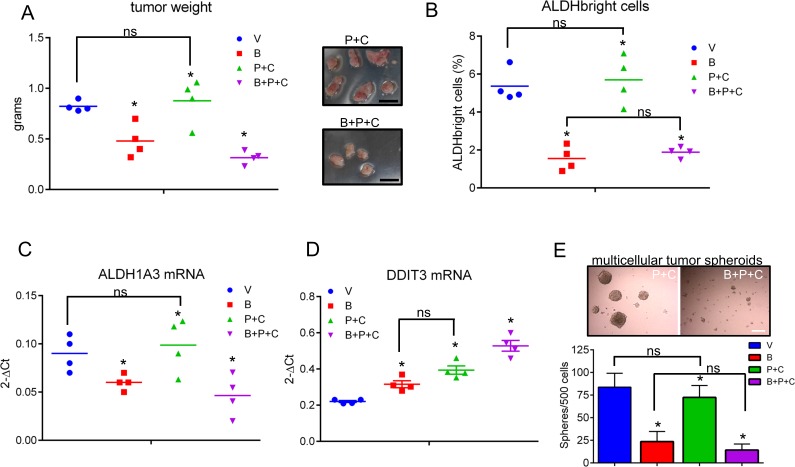
Butein treatment affects the ALDH^bright^ cell number *in vivo* and inhibits tumor growth **A**. Average weight of tumors excised from NOD-SCID mice (n=6/group) injected subcutaneously with 3*10^6 MSTO-211H cells and treated intraperitoneally with vehicle (V, 20 %DMSO/80%corn oil), butein (B, 5mpk), pemetrexed + cisplatin (V+P+C: 45mpk + 7mpk, respectively) and butein + pemetrexed+ cisplatin (B+P+C 5mpk+45mpk+7mpk, respectively) after tumor formation. Duplicate experiments. Inset. Representative micrographs of tumors excised from P+C and B+P+C treated mice, respectively, at day 24 p.i. Scale bar: 0.5 inches. **B**. FACS plots show the average percentage of ALDH^bright^ cells from freshly excised and disaggregated tumors. **C**-**D**. Butein-treated tumors exhibit inverse regulation of DDIT3 and ALDH1A3 mRNAs. The levels of DDIT3 and ALDH1A3 mRNAs were assessed by quantitative PCR in freshly excised tumors. Asterisks indicate outliers. **E**. Upper panel. Representative micrographs of 3D clonogenic assays performed with cells derived from the disaggregated tumors of mice treated with V+P+C or B+P+C. Scale bar: 100 μm. Lower panel. Average number of 3D spheroids formed from the disaggregated tumors. Duplicate experiments. Histogram bars represent the mean ± s.e.m. Statistics: * *p* < 0.05; ns=not significant: (*p* > 0.05). One-way analysis of variance with Tukey's *post hoc* corrections-comparing the mean of each group with the mean of every other group.

In order to translate what we observed *in vitro* in an *in vivo* setting, we performed mouse xenograft experiments. Briefly, NOD-SCID mice were injected subcutaneously with 3*10^6 MSTO-211H cells and treated intraperitoneally with vehicle (V: 20% DMSO/80% corn oil), butein (B: 5mpk), pemetrexed + cisplatin (P+C: 45mpk + 7mpk, respectively) and butein + pemetrexed+ cisplatin (B+P+C 5mpk+45mpk+7mpk, respectively). Treatment (4 i.p injections at day 1, 3, 5 and 7) was started when the tumor ≥ 150mm3 in volume (day 0, n=6 mice/group). Weighting of the excised tumors (at day 24) revealed a significant effect of butein, both when administered alone and, more strongly, when combined to P+C (as compared to those excised from the vehicle-treated mice) (*p*<0.05) (Fig. 5A). Additionally, only 4/6 tumors were detectable in the B+P+C treated mice at the time of excision (Fig. 5A, left and right panel). No statistically significant reduction in weight of the P+C treated tumors as compared to the vehicle-treated tumors was observed (Fig. 5A, left panel). Butein treatment synergized with pemetrexed + cisplatin in reducing tumor weight, thus mirroring the chemosensitizing effects observed *in vitro* (Fig. 4C). We postulated that, as observed *in vitro*, the chemosensitizing effect observed *in vivo* should correlate with a change in the number of the ALDH^bright^ cells within the butein treated tumor masses. FACS analysis of tumors disaggregated within one hour from harvesting revealed that the percentage of ALDH^bright^ cells was significantly reduced in the butein treated tumors (*p* < 0.05) (Fig. 5B). Within the same experimental setting, we observed no statistically significant change in the number of ALDH^bright^ cells within the pemetrexed + cisplatin treated tumors, while butein cotreatment caused the ALDH^bright^ cell number to drop significantly and dramatically upon P+C treatment (as compared to the P+C treated mice, p < 0.05, Fig. 5B).

### Butein-treated tumors exhibit inverse regulation of DDIT3 and ALDH1A3 mRNAs and lower levels of ALDH^bright^ cells

**Figure 6:**
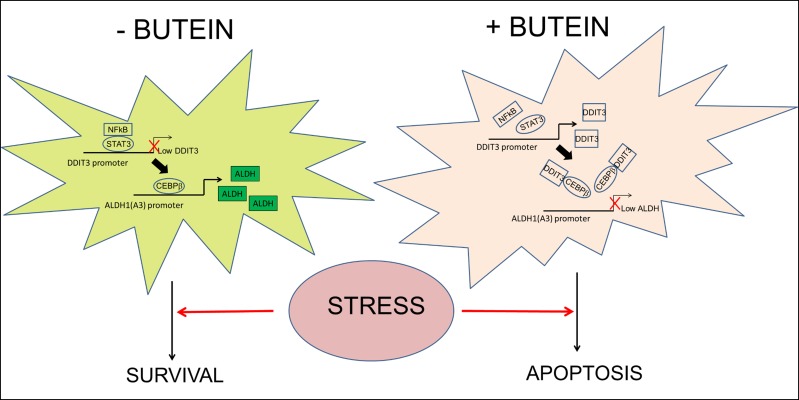
Proposed working model The ALDH^bright^ cells exhibit constitutive activation of the STAT3 pathway which triggers downregulation of DDIT3 mRNA levels both at steady state and upon pemetrexed+cisplatin treatment. Interference with STAT3-NFkB function unlocks expression of DDIT3 in the ALDH^bright^ cells and this reduces the occupancy of ALDH1A3 promoter by CEBPβ thereby lowering ALDH1A3 expression and the intracellular ALDH activity and strongly affecting the survival of the ALDH^bright^ cells to chemotherapy-induced stress.

Quantitative PCR of RNA extracted from pooled tumors (n=4/each group) showed downregulation of the ALDH1A3 mRNA and upregulation of DDIT3 mRNA in all the butein-treated tumors (Fig. 5C-D, respectively). P+C treatment elicited an increase of DDIT3 mRNA as well (Fig. 5D), which however did not correlate with the ALDH^bright^ cell number (Fig. 5B) and with the ALDH1A3 mRNA levels (Fig. 5C). This apparent lack of correlation in the P+C treated tumors was possibly due to the unsorted nature of the samples analyzed, thus reflecting the effect of the DNA damaging agents on the chemosensitive ALDH^low^ cell subpopulations, the predominant cell subpopulation in the excised tumors (Fig. 5B). To verify this possibility, we performed 3D clonogenic assays (a surrogate of tumor relapse) on the same cells and this revealed that the B+P+C treated tumors exhibited a large loss of sphere forming potential as compared to those derived from the P+C treated mice which were mostly unaffected (as compared to the vehicle treated tumor masses, p < 0.05) (Fig. 5E) suggesting a reduced pool of chemoresistant cells in the butein-treated tumors due to targeting of the ALDH^bright^ chemoresistant cell subpopulations. In summary, butein can increase the DDIT3 mRNA levels in the ALDH^bright^ cells and this may rescue the sensitivity of such a cell subpopulation to the chemotherapy-induced stress both *in vitro* and *in vivo* (Fig. 6).

## DISCUSSION

In this work, by exploiting Malignant Pleural Mesothelioma as an experimental model of tumor chemoresistance [4, 41], we demonstrated that a specific ALDH isoform, namely ALDH1A3, is enriched in chemoresistant mesothelioma cell subpopulations purified from multiple sources. This observation echoes what others have observed in breast, melanoma, non-small –cell lung-cancers and mesenchymal-type glioma [14, 18, 19, 30, 42]. Here we can add that expression of ALDH1A3 impinges on the tolerance to stress-induced chemotherapy *in vitro* and *in vivo*. In line with this function, we show that this process is modulated by factors known to play a role in cancer chemoresistance [34, 43-47]. In facts, among the factors shown here to modulate ALDH1A3 expression, C/EBPβ and STAT3 have already emerged as prognostically relevant modulators of the glioma mesenchymal phenotype [31, 48] and NFkB was shown to be constitutively active in a number of tumors [32], including MPM, where its constitutive activity may results from the asbestos-induced chronical inflammation [49]. Additionally, DDIT3 was identified in a TCGA worst-prognosis signature (TWPS) comprising 884 genes differentially expressed in worst versus best prognosis gliomas [31] and low DDIT3 levels may be prognostically relevant for MPM [50]. Notably, low DDIT3 levels are a requirement for RAS-mediated cell transformation [51].

It is very likely that the function of the STAT3/NFkB axis here described does not influence only the ALDH1A3 promoter and may involve modulation of additional CEBPβ-dependent promoters. Additionally, cells endowed with high cytoplasmic levels of DDIT3 exhibited modulation of complex and novel transcriptional targets encompassing both activation and repression functions, when challenged with stress stimuli [52]. Thus, the survival of the ALDH^bright^ cells may rely on additional pathways perturbed by the reciprocal modulation of DDIT3/CEBPβ activity, not addressed here. While this can be a limitation of the present work, it does not detract from the central role played by ALDH1A3 in contributing resistance of the ALDH^bright^ cells to stress. In line with this, the fact that knocking down of ALDH1A3 did not increase the effect of butein strongly suggests that modulation of ALDH1A3 activity is a main target mechanism of this compound.

Here we do not address how the ALDH1A3 expression contributes stress tolerance to the ALDH^bright^ cells. The contribution of ALDH1A3 to survival is probably complex and results from multiple mechanisms. For example, enriched expression for ALDH1A3 was shown to confer specific metabolic features to glioma “stem-like” cells [19]. In line with our observations showing that ALDH1A3 is STAT3 target, it is intriguing to observe how the metabolic phenotype of the ALDH1A3 expressing glioma cells, consisting of aerobic glycolysis, is similar to that of cells expressing a transforming version of the STAT3 protein with increased nuclear retention and transcriptional activity [19, 53]. ALDH1A3 is also very important for the production of retinoic acid (RA) [54, 55]. We may speculate that the ALDH^bright^ cells (enriched for ALDH1A3 expression/activity) may indeed produce retinoic acid (RA) metabolites, the latter acting as paracrine signaling molecules. RA may signal to adjacent cells and confer protumorigenic properties. Relevant to this, Marcato and Lee's groups have demonstrated that ALDH1A3-mediated modulation of RA-target genes contributes *in vivo* protumorigenic properties including the transcription of the MUC4 oncogene [20], to MDA-MB-231 breast cancer cells. Last but not least, our observations suggest that butein-mediated downregulation of ALDH1A3 is pro-apoptotic and this effect is much increased in presence of pemetrexed + cisplatin mediated DNA damage. Interestingly, involvement in the DNA damage response was recently identified as a function of ALDH1A1 in breast, prostate and ovary cancer cells [12, 56, 57]. The downregulation of ALDH1A3 may thus amplify a DNA damage response, consistent with the chemosensitizing effects observed *in vivo*.

On a wider perspective, low DDIT3 levels in the ALDH^bright^ cells may promote unperturbed CEBPβ activity during stress. CEBPβ is a modulator of the mesenchymal phenotype and interference with CEBPβ function (in absence of adipogenic or chondrocyte commitment stimuli), is known to negatively affect the undifferentiated state of mesenchymal precursors [58]. Relevant to this, we have shown that the ALDH^bright^ cells exhibit mesenchymal properties such as expression of mesenchymal markers and hypermigratory features [4] and ALDH activators were shown to increase expression of mesenchymal markers and multipotency of salivary gland precursors [59]. Thus, we may speculate that keeping DDIT3 levels low in stress conditions would allow the ALDH^bright^ cells to survive, to transmit protumorigenic stimuli to adjacent cells and to maintain a specific, possibly differentiation related, metabolic cell identity.

For the *in vivo* studies, we have exploited butein, already known to us for counteracting chemoresistance of MPM cells *in vivo* and *in vitro* [35]. To note, we show here that the 3D clonogenicity of the cells derived from butein-treated excised tumors is strongly reduced. This suggests the possibility that butein may exhaust the chemoresistant cell pool within treated tumors and thus may prevent or strongly delay tumor relapse. This has some translational relevance especially if we consider that butein was shown to not affect normal, untransformed cell lines and to not shorten survival of non-tumor bearing mice [35]. Last, our observations might likely be of interest for other neoplastic diseases characterized by STAT3-NFkB activation and chemoresistance.

## MATERIALS AND METHODS

### Cell lines and culture conditions

The human MPM cell lines MSTO-211H, H-28, H-2052 were from ATCC (Manassas, VA, USA). H-2591, H-2818, H-2595, H-2373, H-2461, HP-1, H-2596 were obtained as described elsewhere [60]. All the cell lines were Mycoplasma free and used at passages 2-6 from thawing. Cells were cultured as monolayers at 37 °C and 5% CO_2_ in DMEM/F12+GLUTAMAX supplemented with 10% non-heat inactivated FBS (fetal bovine serum) (Life Technologies, Gran Island, NY USA). For drug treatments, cells were exposed for the indicated length of time to butein, cisplatin and pemetrexed. Afterthat growth medium was changed with a drug free medium and cells allowed to growth for the additional time indicated in each legend.

### Reagents

Pemetrexed, cisplatin and butein (Selleckchem, Texas, USA) were dissolved according to the manufacturer's instructions.

### Retroviral transduction, promoter reporter and luciferase assay

The shRNA containing vectors targeting ALDH1A3, ALDH1A1, ALDH2 and the scrambled non targeting control vector were previously described [14]. The viral vectors were transfected into 293T packaging cells using Lipofectamine 2000 (Invitrogen, Life Technologies, Grand Island, NY USA) according to the manufacturer's instructions. 48hours later the virus-containing supernatants were filtered (0.45 μM) and used to infect the recipient MPM cell lines. Where possible, the infected cells were selected by puromycin (1 μg/ml every 48 h for 1 week). CHOP promoter/pmCherry-1(#36035, Addgene, Cambridge, MA) was transfected into MPM cells using Lipofectamine 2000 (Life Technologies) according to the manufacturer's instructions. To evaluate the ALDH1A3 promoter transcriptional activity, we used a Luciferase reporter construct (SwitchGear, Active Motif Carlsbad, CA), transfected into cells, according to the manufacturer's instructions. Luciferase activity was measured using the Light Switch Assay reagent (Active Motif, Carlsbad, CA, USA.)

### RNA interference

STAT3, CEBPβ, NFkB (p65-RelA) targeting siRNA (TRILENCER-27 siRNAs) were from Origene Technologies (Rockwille, MD, USA) and were transfected with siTRAN siRNA transfection reagent following manufacturer's instructions. Cells were harvested at 24hrs (for RNA extraction) or 48hrs (for protein studies)

### ALDH activity assay and cell sorting

ALDEFLUOR kit (Stem Cell Technologies, Vancouver, Canada) was used according to the manufacturer's instructions. ALDH-positive cells were defined as the cells that displayed greater fluorescence compared with a control staining reaction containing the ALDH inhibitor, DEAB (diethylaminobenzaldehyde), upon addition of the synthetic ALDH substrate BAAA. Cell sorting was performed using a FACSAria flow cytometer (Becton Dickinson, Mississauga, Canada). Dead cells positive to SYTOX Red Dead Cell Stain (Life Technologies, Grand Island, NY) were excluded.

### Colony forming assay (CFA)

MPM cell lines were grown to 70% confluence and pulse- treated with the indicated drugs or transfected as indicated. 16hrs later, cells were detached and seeded at 500-1500 cells/well into 6-well dishes in drug-free media (2ml medium /well). Fresh medium (25%) was added every three days. Colonies were stained with crystal violet (SIGMA) and colonies (>50 cells) counted after 7- 14 days (this wide range reflects differences in the proliferation of the colonies for each MPM cell line). For 3D clonogenic assays, the cells were plated in anchorage independent and serum free conditions in DMEM-F12/1:1 + Glutamax supplemented with BSA and EGF(10ng/ml) and FGF2(10 ng/ml) (Life Technologies) as previously described [61].

### RNA extraction and cDNA synthesis and gene expression

Total RNA was extracted using the RNAeasy minikit (QIAGEN). The first-strand cDNA was synthesized with the High Capacity RNA-to cDNA kit, (Applied Biosystems). Gene expression was measured by real-time PCR using the SYBRGreen dye (Applied Biosytems) on a Step One instrument (Applied Biosytems). Specific primers for ALDH isoforms were described previously [14]. DDIT3 primers were: forward: -GGAAACAGAGTGGTCATTCCC; reverse: CTGCTTGAGCCG-TTCATTCTC. PPIA and HPRT were used as endogenous control and were described previously [62].

### Immunofluorescence microscopy

Briefly, the cells were fixed and permeabilized in paraformaldehyde/methanol, non-specific binding blocked with PBS containing 1% BSA for 1 hour at room temperature and labeled with a mouse monoclonal anti-DDIT3 (Abcam, Cambridge, UK). The secondary antibody was an anti-mouse IgG Texas red (Abcam). Cell nuclei were visualized by 4′,6-diamidino-2-phenylindole (DAPI) staining. For counting the mCHERRY positive cells, a minimum of 8 fields (containing ≥40 cells) was counted in duplicate experiments. Quantification of fluorescence was performed in ImageJ® and the number of cells with a mean fluorescent intensity (MFI) above the threshold (set on untransfected cells) was reported were indicated.

### Apoptosis detection

Dead cells were measured by FACS analysis after SYTOX Dead Cell Stain-labeling (Life Technologies, Gran Island, NY USA), according to the manufacturer's instructions. The Human Apoptosis Antibody Array (R&D, Minneapolis, MN, USA) was used to simultaneously detect the relative expression of 35 apoptosis-related proteins.

### Cell lysis, immunoprecipitation and western blotting

Briefly, cells were lysed in cell lysis buffer (50mM Tris-HCl (pH 8), 0.5% IGEPAL AC-630, 150 mM NaCl, 1mM EDTA, and 10% glycerol, supplemented with protease and phosphatase inhibitors, to generate total cell extracts. For the immunoprecipitation studies the following antibodies were used: mouse anti-CHOP (Abcam, Cambridge, UK), mouse anti-STAT3 (Santa Cruz Biotechnology, CA, USA), rabbit anti-phosphoSTAT3(tyr705)(Cell Signaling), mouse-anti-CEPBβ, rabbit-anti-NFKB (Santa Cruz Biotechnology). For the western blotting: rabbit anti- CHOP, rabbit anti-CEPBβ (Cell Signaling, Danvers, MA, USA), goat anti-ALDH1A3 (Santa Cruz Biotechnology), rabbit anti-p-STAT3 (Cell Signaling), rabbit anti-STAT3 (Cell Signaling), mouse anti-ALDH2 (Abnova, Walnut, CA, USA), mouse anti-ALDH1 (this antibody mainly recognizes ALDH1A1) (BD Biosciences, NJ, USA), rabbit anti-ALDH1A3 (Abgent, San Diego, CA, USA) were used. Rabbit anti-ACTIN staining (Santa Cruz Biotechnology) or mouse anti-GAPDH (Pierce, Rockford, IL, USA) were used as a loading control. For the chemiluminescent detection of the secondary antibodies, a Western Bright ECL HRP substrate (ADVANSTA, Menlo Park, CA,) was used. Please note that for the western blotting of lysates from purified cell subpopulations, a supersensitive detection reagent was used (WesternBright Sirius HRP substrate) given the low amount of material available. Densitometry was performed on scanned western blotting images using the ImageJ^®^ software. Relative intensity for each protein band was reported after normalization to its loading control, where indicated in the figure legend.

### DNA affinity precipitation assay

Briefly, cells were collected in cold PBS/2 mM EDTA and lysed in DAPA lysis buffer (50 mM Tris-HCl pH 8, 0.5% IGEPAL-AC-630, 100 mM NaCl, 1 mM EDTA, 1 mM MgCl_2_ and 10% glycerol), supplemented with protease and phosphatase inhibitors to generate total cell extracts. Cell extracts were diluted three times in IP buffer (20 mM Tris-HCl pH 8, 10 mM NaCl, 0.5 mM EDTA, 0.5 mM MgCl_2_ and 10% glycerol) with protease and phosphatase inhibitors and incubated with the 5′-biotinylated DNA oligonucleotides (2.5 μg/0.5 mg cell lysate) complexed to streptavidin-agarose magnetic beads in IP buffer at 4°C for 2 h. The oligo-beads complex was washed 5 times with washing buffer (10 mM Tris-HCl pH 8, 0.5% IGEPAL-AC-630, 175 mM NaCl, 0.25 mM MgCl_2_ and 5% glycerol) and eluted with Laemmli buffer at 65C for 5 minutes. The sequence of the oligonucleotide isbiotin-5-TCTTCATTTCCAGGAGGTGAAA-3. As a control 10 μg of competitor non-biotin-labeled oligonucleotide were preincubated with the nuclear extract before adding the biotin-labeled one as described above.

### Chromatin immunoprecipitation (CHIP)

SimpleChIP® Enzymatic Chromatin IP Kit Magnetic Beads(Cell Signaling) was used according to the manufacturer's instructions except that the supernatant of the digestion reaction (Micrococcal Nuclease) was not discarded and used for the immunoprecipitation cocktail Briefly, 150 μg of crosslinked/sonicated chromatin was incubated with antibody overnight at 4°C. Antibodies used were as follows: mouse anti-STAT3 and rabbit anti-CEPBβ (Santa Cruz Biotechnology, Santa Cruz, CA); anti-pSTAT3(Tyr705), rabbit-anti-NFkB (Cell Signaling) and the negative control Normal Rabbit IgG samples (Cell Signaling). CHIP Grade Protein G Magnetic Beads were added to the samples after BSA blocking and incubated for 4 h at 4°C with rotation. After several washes (low salt and high salt), complexes were eluted and DNA cross-linking reversed. DNA was purified using Spin Columns. Quantitative PCR for CHIP analysis was performed as indicated before, by using SYBR green (Applied Biosystems). Fold-change enrichment (relative to a 2% input chromatin) was calculated using the formula: Percent Input = 2% x 2^(C[T] 2%Input Sample – C[T] IP Sample). The specific primers for the CEBPβ binding site in the ALDH1A3 promoter (chr15: 101419959-101419973) and for the STAT3 binding site in the DDIT3 promoter (chr12: 57911053-57911074) and the “off target” controls (Epitect CHIP primers) were commercially available (Qiagen, Valencia, CA).

### ALDH1A3 and DDIT3 promoter analysis

Promoter analysis was performed using the MatInspector software (http://www.genomatix.de) and MATCH^TM^, BIOBASE. A sequence 2 kb upstream and 2 kb downstream from the transcription start site was analyzed for the presence of putative binding sites for each TF.

### Animal studies

All animal work was performed in accordance with NYU guidelines and upon IACUC approval. Suspensions of 3 × 10^6^ MSTO-211H cells were injected subcutaneously. in PBS1X into 5-weeks-old male NOD/SCID mice (Charles River, Italy). Body weight and clinical signs of the mice were determined every 3 days. When tumor volume ≥ 150mm^3^, mice were randomized and treated intraperitoneally with vehicle (V, 20% DMSO/80% corn oil), butein (B, 5mpk), pemetrexed + cisplatin (P+C: 45mpk + 7mpk, respectively) and butein + pemetrexed+ cisplatin (B+P+C 5mpk+45mpk+7mpk, respectively). Treatment (4 i.p injections at day 1,3,5,7) started when the tumor ≥ 150mm3 in volume (day 0, n=6 mice/group).

### Statistical analysis

One-way analysis of variance with Tukey's *post hoc* corrections-comparing the mean of each group with the mean of every other group or Student's *t*-test (comparing each sample to its control or, when indicated, to other samples within the same group). Statistical significance was defined as *p* < 0.05 where indicated. Except when indicated in the legend, all the data were from at least 3 biological replicate experiments. The GraphPad software was used for all the statistics.

## SUPPLEMENTARY MATERIAL AND FIGURES


