# Supplementary material for: A STAT3-NFkB/DDIT3/CEBPβ axis modulates ALDH1A3 expression in chemoresistant cell subpopulations
Source: Oncotarget. 2015 Mar 30;6(14):12637–53. doi: 10.18632/oncotarget.3703 (PMC4494963; doi:10.18632/oncotarget.3703)
Supplement: Supplementary file 1 [file oncotarget-06-12637-s001.pdf]

A STAT3-NFkB/DDIT3/CEBPβ axis modulates ALDH1A3 expression in chemoresistant cell subpopulations

Supplementary Material

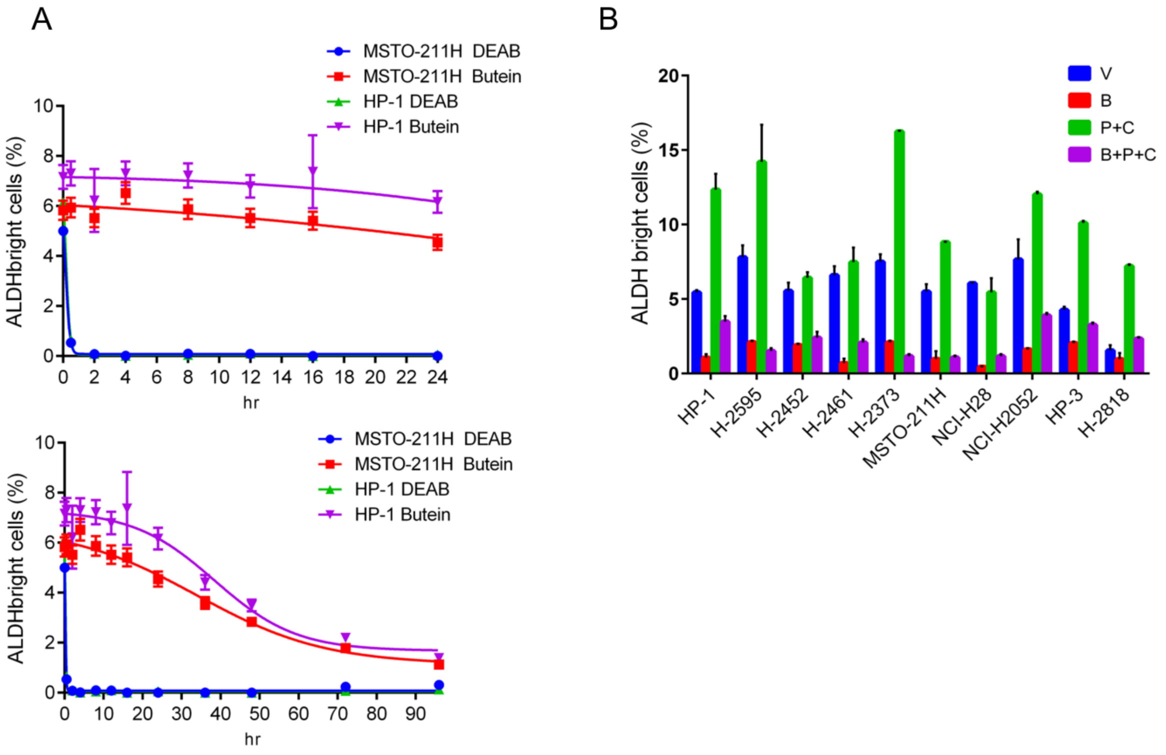

Suppl. Fig. 1: **Butein affects the ALDHbright cell number in MPM cell cultures**. **A**.**Butein does not inhibit the ALDH enzyme.** Graph showing the number of $ALDH^{bright}$ cells in time (0-90h) after treatment with butein or DEAB (diethylaminobenzaldehyde, 5μM), a specific ALDH inhibitor. Average and s.e.m of triplicate experiments. **B.** Histograms show the percentage of $ALDH^{bright}$ cells in the indicated MPM cell cultures treated for 24hrs with vehicle (V: DMSO 0.05%) and Butein (B: 18μM), alone or in combination with pemetrexed + cisplatin (P+C: 10 μM + 5 μM, respectively) and stained for ALDH activity at 96hrs. The percentage of $ALDH^{bright}$ cells was determined over the same cells treated with a specific ALDH inhibitor (DEAB) immediately after adding the ALDH substrate (BAA). Average + s.e.m of triplicate experiments.

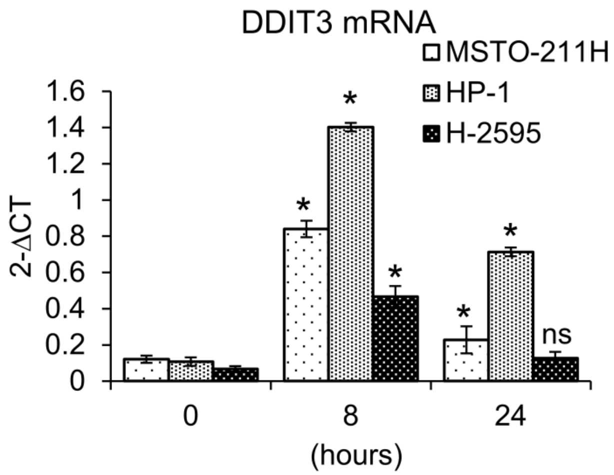

Suppl. Fig. 2: Butein modulates the DDIT3 mRNA levels. Quantitative PCR**.** DDIT3 mRNA levels of three representative MPM cell lines treated with butein (18μM) for the indicated times. Histogram bars represent the mean ± s.e.m of ≥ three experiments. Statistics: p<0.05. Statistics: * p<0.05; ns=not significant: (p>0.05). Student's t-test (comparing each sample to its control at 0hrs)

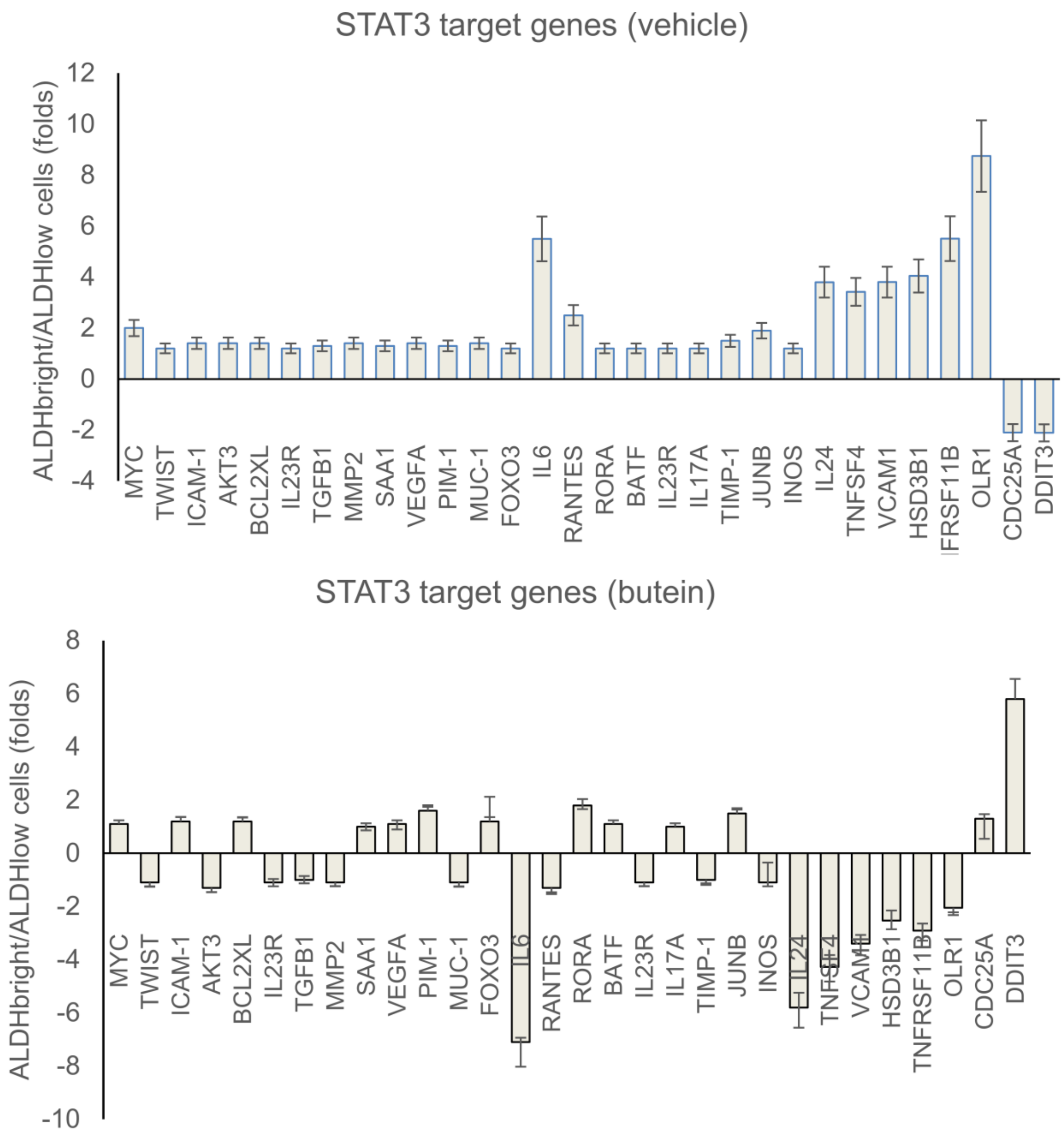

Suppl. Fig. 3: **Butein affects the levels of STAT3 target genes in ALDHbright and ALDHlow cells**. mRNA levels of multiple STAT3 target genes in MSTO-211H ALDH^{bright} vs ALDH^{low} cells, upon treatment with vehicle (upper) or butein (18μM) (lower) for 24hrs. Histogram bars show the average + s.e.m of duplicate experiments.
